# Alternating Electric Fields (TTFields) Activate Ca_v_1.2 Channels in Human Glioblastoma Cells

**DOI:** 10.3390/cancers11010110

**Published:** 2019-01-18

**Authors:** Eric Neuhaus, Lisa Zirjacks, Katrin Ganser, Lukas Klumpp, Uwe Schüler, Daniel Zips, Franziska Eckert, Stephan M. Huber

**Affiliations:** 1 Department of Radiation Oncology, University of Tübingen, Hoppe-Seyler-Str. 3, 72076 Tübingen, Germany; eric.neuhaus@med.uni-tuebingen.de (E.N.); lisa-zirjacks@web.de (L.Z.); katrin.ganser@med.uni-tuebingen.de (K.G.); lukas.klumpp@med.uni-tuebingen.de (L.K.); daniel.zips@med.uni-tuebingen.de (D.Z.); franziska.eckert@med.uni-tuebingen.de (F.E.); 2 Institute of Applied Physics, University of Tübingen, Auf der Morgenstelle 10, 72076 Tübingen, Germany; uwe.schueler@uni-tuebingen.de; 3 German Cancer Consortium (DKTK) partnersite Tübingen, German Cancer Research Center (DKFZ), Im Neuenheimer Feld 280, 69120 Heidelberg, Germany

**Keywords:** glioma, alternating electric field therapy, Ca^2+^ signaling, programmed cell death, clonogenicity, L-type Ca^2+^ channel, benidipine

## Abstract

Tumor treating fields (TTFields) represent a novel FDA-approved treatment modality for patients with newly diagnosed or recurrent glioblastoma multiforme. This therapy applies intermediate frequency alternating electric fields with low intensity to the tumor volume by the use of non-invasive transducer electrode arrays. Mechanistically, TTFields have been proposed to impair formation of the mitotic spindle apparatus and cytokinesis. In order to identify further potential molecular targets, here the effects of TTFields on Ca^2+^-signaling, ion channel activity in the plasma membrane, cell cycle, cell death, and clonogenic survival were tested in two human glioblastoma cell lines in vitro by fura-2 Ca^2+^ imaging, patch-clamp cell-attached recordings, flow cytometry and pre-plated colony formation assay. In addition, the expression of voltage-gated Ca^2+^ (Ca_v_) channels was determined by real-time RT-PCR and their significance for the cellular TTFields response defined by knock-down and pharmacological blockade. As a result, TTFields stimulated in a cell line-dependent manner a Ca_v_1.2-mediated Ca^2+^ entry, G_1_ or S phase cell cycle arrest, breakdown of the inner mitochondrial membrane potential and DNA degradation, and/or decline of clonogenic survival suggesting a tumoricidal action of TTFields. Moreover, inhibition of Ca_v_1.2 by benidipine aggravated in one glioblastoma line the TTFields effects suggesting that Ca_v_1.2-triggered signaling contributes to cellular TTFields stress response. In conclusion, the present study identified Ca_v_1.2 channels as TTFields target in the plasma membrane and provides the rationale to combine TTFields therapy with Ca^2+^ antagonists that are already in clinical use.

## 1. Introduction

Tumor Treating Fields (TTFields) therapy, developed by the company NovoCure (Haifa, Israel) is a new modality of anti-cancer therapy which has been FDA-approved for newly diagnosed and recurrent glioblastoma multiforme and is currently evaluated for several other tumor entities. TTFields apply alternating intermediate-frequency (200 kHz) sine wave-electric fields with low field strength (1–3 V/cm) to the tumor with the help of non-invasive ceramic transducer electrode arrays (for glioblastoma patients mounted on the shaved scalp). TTFields were usually administered 20–24 h/day for several months [1] concomitantly to temozolomide maintenance therapy after surgical resection of the glioblastoma and subsequent radiochemotherapy with temozolomide. A phase III clinical trial on recurrent glioblastoma comparing TTFields monotherapy with physician’s choice chemotherapy indicates that the efficacy of TTFields is similar to chemotherapy with improved toxicity profile [1]. The 6-year-follow-up of a prospective randomized multicenter phase III trial on newly diagnosed glioblastoma comparing standard temozolomide maintenance therapy with standard therapy plus TTFields electrotherapy indicates a significant improvement of progression-free and overall survival by TTFields [2]. Importantly, the adverse side effects of TTFields therapy are restricted to mild to moderate skin rash beneath the transducer arrays [1,3]. Combined these clinical data indicate that TTFields electrotherapy is an effective and safe treatment modality for glioblastoma patients increasingly offered to patients in good performance status willing to wear the electrode arrays.

TTFields reportedly target mitosis and cytokinesis [4,5,6,7]. Mechanistically, TTFields have been proposed to align proteins possessing high intramolecular dipole moments such as tubulin dimers [6] and septins [7] which counteracts proper formation of the mitotic spindle apparatus and positioning of the cytokinetic furrow, respectively. In addition, dielectrophoretic forces in areas of non-uniform TTFields as proposed to occur at the cytokinetic furrow within the dividing cells have been postulated to trap polar macromolecules and organelles and to impair their symmetrical distribution between daughter cells. As a consequence, TTFields induce aberrant metaphase exit, improper chromosome segregation and mitotic catastrophe eventually resulting in cell death [8]. 

By inhibiting mitosis and cytokinesis, TTFields in particular target fast proliferating cells which results in a certain tumor specificity. Not surprisingly, besides glioblastoma, several other tumor entities such as skin, breast, pancreatic, lung, and ovarian cancer have been reported to react on TTFields in vitro [5,6,7,9,10,11,12,13,14], in preclinical in vivo models [5,9,12,13,15,16,17] or in clinical pilot studies [9,18,19,20]. Notably, best, i.e., most effective TTFields frequency and field strength differ between the different tumor entities with a best frequency of 200 kHz for glioblastoma [4,9,21].

Beyond impairment of mitosis and cytokinesis, TTFields reportedly lower metastatic spread of tail vein-injected B16 melanoma cells [15], and sensitize to chemo- [10], targeted [22], or radiation therapy [23]. The latter probably results from TTFields-mediated delay in DNA double strand break repair [23] possibly via downregulation of BRCA1 signaling [14]. Importantly, immunosuppression by dexamethasone seems to attenuate the response of glioblastoma patients to TTFields therapy [24] illustrating the function of the immune system in the tumor control by TTFields. Combined, these data point to a complex biological response to TTFields therapy.

Therefore, the present study aimed to identify molecular TTFields targets beyond the proposed mitotic spindle apparatus or the cytokinesis furrow. Such novel targets might be used as markers for patient stratification in terms of therapy personalization. In addition, the identification of novel molecular pathways that are triggered by TTFields and that confer resistance against the electric field therapy bears the great chance for pharmacological intervention and amplification of TTFields efficacy.

Our study placed emphasis on Ca^2+^ signaling and Ca^2+^-regulated cellular processes such as cell cycle or cell death since alternating electrical fields have been demonstrated to interfere with intracellular Ca^2+^ signals. For instance, voltage-activated Ca^2+^ (Ca_v_) channels in the electrosensory organs of sharks are involved in perception of changes in the environmental electrical fields [25]. Likewise in mammalian cells, experimental or environmental alternating electric fields have been demonstrated to evoke intracellular Ca^2+^ signals [26,27,28,29,30,31,32] that may involve in particular L-type Ca_v_ channels in the plasma membrane (for review see [33,34,35]). The alpha subunits of the L-type Ca^2+^ channels Ca_v_1.1, Ca_v_1.2, Ca_v_1.3, and Ca_v_1.4 are encoded by the CACNA1S, -1C, -1D, and 1F gene, respectively. While Ca_v_1.1 is mainly expressed in the skeletal muscle and Ca_v_1.4 restricted to retina, Ca_v_1.2 and Ca_v_1.3 are expressed in most excitable cells. In brain, Ca_v_1.2 and Ca_v_1.3 are found in the dendrites and soma of neurons where among others they are involved in the excitation-transcription coupling [36]. Notably, Ca_v_ channels are also expressed by glioblastoma cells [37]. Potential downstream targets of Ca_v_ are Ca^2+^- activated high conductance BK_Ca_ [38,39,40,41] and intermediate conductance IK_Ca_ (K_Ca_3.1, SK4) K^+^ channels [42,43,44] that have been reported to contribute to cell migration or therapy resistance of glioblastoma cells. Hence, here we analyzed TTFields-evoked modulation of cytosolic free Ca^2+^ concentration (_free_[Ca^2+^]_i_) and Ca^2+^-dependent ion channel activity in the plasma membrane in individual human glioblastoma cells with the help of a single cell TTFields applicator disclosing a TTFields-stimulated Ca^2+^ entry that involves Ca_v_1.2 channels. In a second step, effects of TTFields on cellular DNA content, mitochondrial membrane potential (ΔΨ_m_) and colony formation were analyzed in dependence on pharmacological Ca_v_ blockage with the help of a TTFields cell culture applicator in order to assess cell cycle distribution, asymmetric cell division, cell death, triggering of intrinsic apoptosis, and clonogenic survival.

## 2. Materials and Methods

### 2.1. Cell Culture and Transfection

Human T98G (obtained from American Type Cell Culture Collection (ATCC, Manassas, VA, USA) and U251 (kindly provided by Dr. Luiz Penalva, San Antonio, TX, USA) glioblastoma cells were grown in 10% fetal calf serum (FCS)-supplemented RPMI-1640 (T98G) or DMEM (4500 mg glucose/L, U251) medium. For CACNA1C knock-down, exponentially growing U251 and T98G cells were reversely transfected with a mixture of three Stealth siRNAs (Thermo Fischer Scientific, Waltham, MA, USA) specific for human CACNA1C (HSS187849, HSS187850, HSS187851) or with nt siRNA (*Silencer*® Select Negative Control No. 1 siRNA, #4390844, Ambion™, Thermo Fischer Scientific). Detached T98G and U251 cells (250,000 in 2.5 mL RPMI-1640/10% FCS and DMEM (4500 mg glucose/L)/10% FCS medium, respectively) were added to 500 µL of pre-incubated (20 min at room temperature) Opti-MEM medium containing RNAiMAX lipofectamine (6 µL, Invitrogen Life Technologies, Carlsbad, CA, USA) and siRNA (25 nM final concentration).

### 2.2. Fura-2 Fluorescence Imaging of Cytosolic Free Ca^2+^ Concentration (_free_[Ca^2+^]_i_)

Fluorescence measurements were performed at 37 °C using an inverted phase-contrast microscope (Axiovert 100; Zeiss, Oberkochen, Germany). Fluorescence was evoked by a filter wheel (Visitron Systems, Puchheim, Germany)-mediated alternative excitation at 340/26 or 387/11 nm (AHF, Analysentechnik, Tübingen, Germany). Excitation and emission light was deflected by a dichromic mirror (409 nm beam splitter, AHF) into the objective (Fluar 40×/1.30 oil; Zeiss) and transmitted to the camera (Visitron Systems), respectively. Emitted fluorescence intensity was recorded at 587/35 nm (AHF). Excitation was controlled and data acquired by Metafluor computer software (Universal Imaging, Downingtown, PA, USA). The 340/380-nm fluorescence ratio was used as a measure of _free_[Ca^2+^]_i_. Control, CACNA1C- or nt siRNA-transfected T98G and U251 cells (48 h after transfection) were incubated with fura-2/AM (2 µM for 30 min at 37 °C; Molecular Probes, Goettingen, Germany) in RPMI-1640/10% FCS and DMEM/10% FCS medium, respectively. _free_[Ca^2+^]_i_ was recorded at 37 °C during superfusion with Ca^2+^-containing NaCl solution (in mM: 125 NaCl, 32 HEPES, 5 KCl, 5 d-glucose, 1 MgCl_2_, 1 CaCl_2_, titrated with NaOH to pH 7.4), with Ca^2+^-free NaCl solution (in mM: 125 NaCl, 32 HEPES, 5 KCl, 5 d-glucose, 1 MgCl_2_, 0.6 EGTA, titrated with NaOH to pH 7.4), or with Ca^2+^-containing NaCl solution further containing the L-, N-, T-type Ca^2+^ channel blocker benidipine or the L-type inhibitor nifedipine (both 1 µM, Sigma-Aldrich, Taufkirchen, Germany) before, during and after application of TTFields (200 kHz, 0–2.5 V/cm).

### 2.3. Patch Clamp Recording

Currents of semi-confluent T98G and U251 cells were elicited by 33 voltage square pulses (700 ms each) delivered in 5 mV increments from 0 mV holding potential to voltages between −80 mV and +80 mV and recorded at 37 °C in cell-attached, voltage-clamp mode by an EPC-9 amplifier (Heka, Lambrecht, Germany) using Pulse software (Heka) and an ITC-16 Interface (Instrutech, Port Washington, NY, USA).

Clamp voltages refer to the intracellular face of the plasma membrane. Flow of positive charge out of the cells (or the counter flow of anions) is defined as positive current and depicted as upward deflection of the current tracings. Cells were superfused at 37 °C with Ca^2+^-containing NaCl solution (see above). Borosilicate glass pipettes (4–6 MΩ pipette resistance; GC150 TF-10, Clark Medical Instruments, Pangbourne, UK) manufactured by a microprocessor-driven DMZ puller (Zeitz, Augsburg, Germany) filled with Ca^2+^-containing NaCl solution (see above) were used in combination with a STM electrical micromanipulator (Lang GmbH and Co KG, Hüttenberg, Germany). Macroscopic cell-attached currents were analyzed by averaging the currents between 100 and 700 ms of each square pulse. In addition to macroscopic cell-attached currents unitary current transitions were characterized for single channel conductance and open probability (P_o_). The latter was estimated by subtracting the zero current (i.e., the current at no apparent unitary current transition) at a given clamp voltage from the averaged macroscopic current and by dividing the difference by the amplitude of the unitary current transition and by the apparent number of active channels.

### 2.4. Quantitative RT-PCR 

RNA of control, CACNA1C siRNA-, or nt siRNA-transfected U251 and T98G cells (48 h after transfection) was isolated (NucleoSpin^®^ RNA kit, Machery-Nagel, Düren, Germany) and reversely transcribed and CACNA1A, -1B, -1C, -1D, -1E, -1G, -1H, -1I, -1S as well as housekeeper β-actin (ACTB)-, pyruvate dehydrogenase beta (PDHB)-, and glyceraldehyde-3-phosphate dehydrogenase (GAPDH)-specific fragments were amplified by the use of SYBR Green-based quantitative real-time PCR (1Step RT qPCR Green ROX L Kit, highQu, Kraichtal, Germany, and QuantiTect Primer Assays QT00054152, QT00077042, QT00053480, QT00076657, QT00063994, QT00043043, QT00075159, QT00021126, QT00000833, QT00095431, QT00031227, Qiagen, Hilden, Germany) in a Roche LightCycler^®^480 Instrument (Roche, Mannheim, Germany). mRNA abundances were normalized to the geometrical mean abundance of the three housekeepers. 

### 2.5. Analysis of Cell Cycle, DNA Degradation and Aneuploidy 

Exponentially growing T98G and U251 cells were treated for 5–7 days with TTFields (200 kHz, 1 V/cm) in the presence of benidipine (0 or 1 µM in 0.1% DMSO), trypsinized, washed, and stained (30 min at room temperature) with propidium iodide (PI, Sigma-Aldrich) in RNase-containing phosphate-buffered saline (PBS) further containing 0.1% Na-citrate, 0.1% triton X-100, and PI (10 µg/mL). For cell cycle analysis, DNA amount was recorded by flow cytometry (FACS Calibur, Becton Dickinson, Heidelberg, Germany, 488 nm excitation wavelength) with FL-3 (>670 nm, linear scale) emission wavelength. In addition, DNA content was monitored with FL-2 emission wavelength (585/42 nm, log scale) for determination of sub-G_1_ (DNA degradation) and hyper-G (aneuploidy) populations, respectively. Data were analyzed with the FCS Express 3 software (De Novo Software, Los Angeles, CA, USA). 

### 2.6. Determination of Inner Mitochondrial Membrane Potential (ΔΨ_m_)

TTFields-treated (5–7 days, 200 kHz, 1 V/cm) and benidipine-co-treated (0 or 1 µM in 0.1% DMSO) T98G and U251 cells were trypsinized, washed, and incubated for 30 min at room temperature in Ca^2+^-containing NaCl solution (see above) containing the ΔΨ_m_-specific dye tetramethylrhodamine ethyl ester perchlorate (TMRE, 25 nM, Invitrogen, Karlsruhe, Germany). TMRE-specific fluorescence was measured by flow cytometry with FL-2 (585/42 nm) emission wavelength.

### 2.7. Colony Formation Assay

To test for clonogenic survival, T98G and U251 were pre-plated (600 cells/well in 12-well plates), co-incubated with benidipine (0 or 3 µM in 0.3% DMSO) and subjected to TTFields (200 kHz, 1 V/cm) for 5–7 days and further grown for 10–14 days in the continuous presence of benidipine. Thereafter, clusters of ≥ 50 cells were defined as colonies and counted manually after fixation and Coomassie staining. The plating efficiency was defined by dividing the number of colonies by the number of plated cells. Survival fractions were calculated by dividing the plating efficiency of the TTFields-treated cells by those of the TTFields-untreated vehicle controls.

### 2.8. Statistics

Data are given as means ± standard error (SE). Probability (*p*) of statistical significance was estimated with two-tailed t-test or Welch-corrected two-tailed *t*-test where appropriate. *p* values of *p* ≤ 0.05 (2 samples) or *np* ≤ 0.05 (> 2 samples) was assumed to be significantly different with *n* = number of pair wise comparisons in multiple testing (Bonferroni correction).

## 3. Results

**Figure 1:**
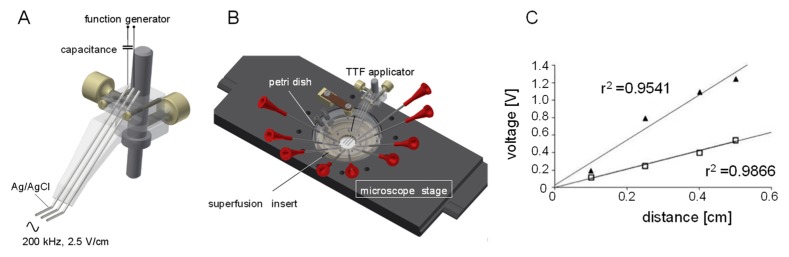
Single cell TTFields applicator. (**A**) Drawing of the applicator. TTFields are applied conductively by two Ag/AgCl electrodes connected via a capacitance (to avoid flow of offset direct current) to a function generator (3rd electrode was originally designed for a parallel real-time 0 V/cm-field strength control but not used). (**B**) Positioning of the TTFields applicator, Petri dish, and superfusion/heating insert at the stage of an inverted microscope. TTFields application and cell recording were performed at 37 °C during continuous superfusion with bath solution. Field strength in the bath solution between both application electrodes at the dish bottom was controlled by the use of two Ag/AgCl recording electrodes. (**C**) Recorded voltages (peak to peak) within the TTFields at different distances. TTFields field strength was adjusted to 2.5 V/cm (closed triangles) and 1 V/cm (open squares) in NaCl solution, respectively. Recorded voltages were fitted by linear regression. The obtained correlation coefficients (r^2^) were r^2^ > 0.9 suggesting a homogeneous distribution of the alternating electric fields between the applicator electrodes.

To identify molecular TTFields targets, a TTFields single cell applicator (Figure 1) was constructed and connected to a function generator. Attached to the stage of an inverted microscope, the TTFields single cell applicator allowed application of electromagnetic sine waves of variable amplitude and frequency to individual cells. TTFields were applied parallel to the plane of the cell layer in a conductive manner via Ag/AgCl electrodes. Here, the only difference to a capacitive TTFields injection (as applied to the patients) is that in the conductive situation possibly biological active Ag ions may accumulate in the cell bathing solution predominantly at the electrode/solution interface. This, however, was prevented by constant superfusion of the cells that guaranteed fast bath solution exchange. The function generator was set to 200 kHz sine waves and the output adjusted to electric field strength of 0.25–2.5 V/cm measured in the bath solution between the two electrodes (Figure 1C).

**Figure 2:**
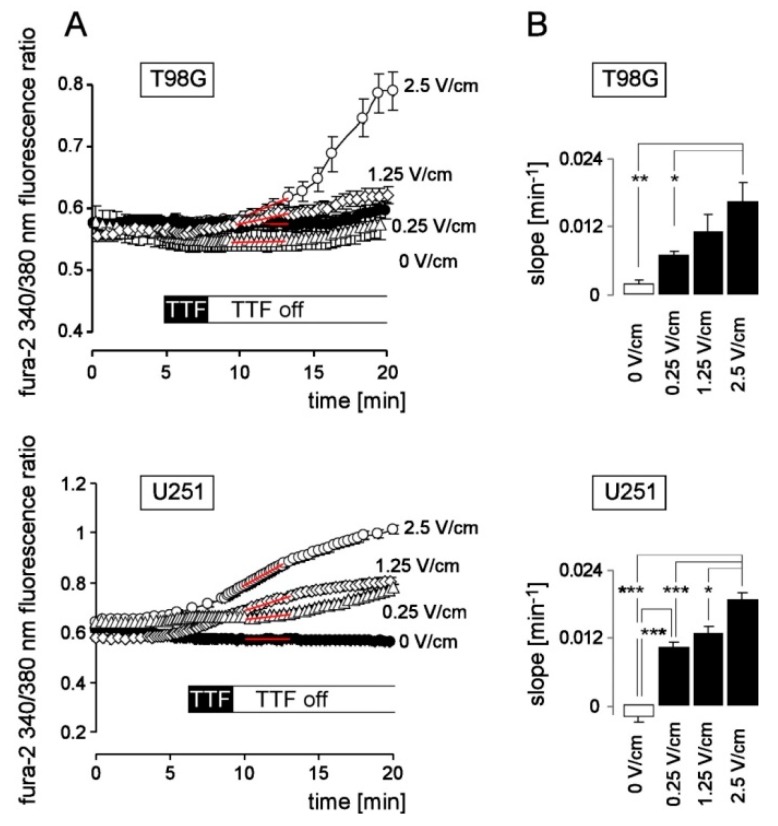
TTFields induce Ca^2+^ signals in U251 and T98G human glioblastoma cells in a dose-dependent manner. (**A**) Time course of mean (±SE; *n* = 8–17) fura-2 340/380 nm fluorescence ratio as a measure of _free_[Ca^2+^]_i_ recorded in T98G (top) and U251 cells (bottom) during superfusion with 1 mM Ca^2+^-containing NaCl-solution before, during and after application of 0 (control), 0.25, 1.25, or 2.5 V/cm TTFields (200 kHz) field strength for 3 min. (**B**) Mean (±SE; *n* = 8–55) slope (as indicated by red lines in (**A**) of the TTFields-induced increase in fura-2 340/380 nm fluorescence ratio as calculated for U251 (left), and T98G (right) cells. *, ** and *** in (**B**) indicate 6*p* ≤ 0.05, 6*p* ≤ 0.01, and 6*p* ≤ 0.001, respectively, (Welch)-corrected t-test and Bonferroni correction for 6 pairwise comparisons.

Since low alternating electric fields have been reported to interfere with intracellular Ca^2+^ signaling (see Section 1) we first assessed TTFields-induced changes in intracellular free Ca^2+^ concentration (_free_[Ca^2+^]_i_) by ratiometric fura-2 Ca^2+^ imaging. As a result, acute application of TTFields to U251 and T98G glioblastoma cells induced a long-lasting increase in _free_[Ca^2+^]_i_ in an electric field intensity (0.25–2.5 V/cm)-dependent manner (Figure 2A,B). In particular, _free_[Ca^2+^]_i_ continued to rise for more than 10 min after switching off the TTFields stimulation.

**Figure 3:**
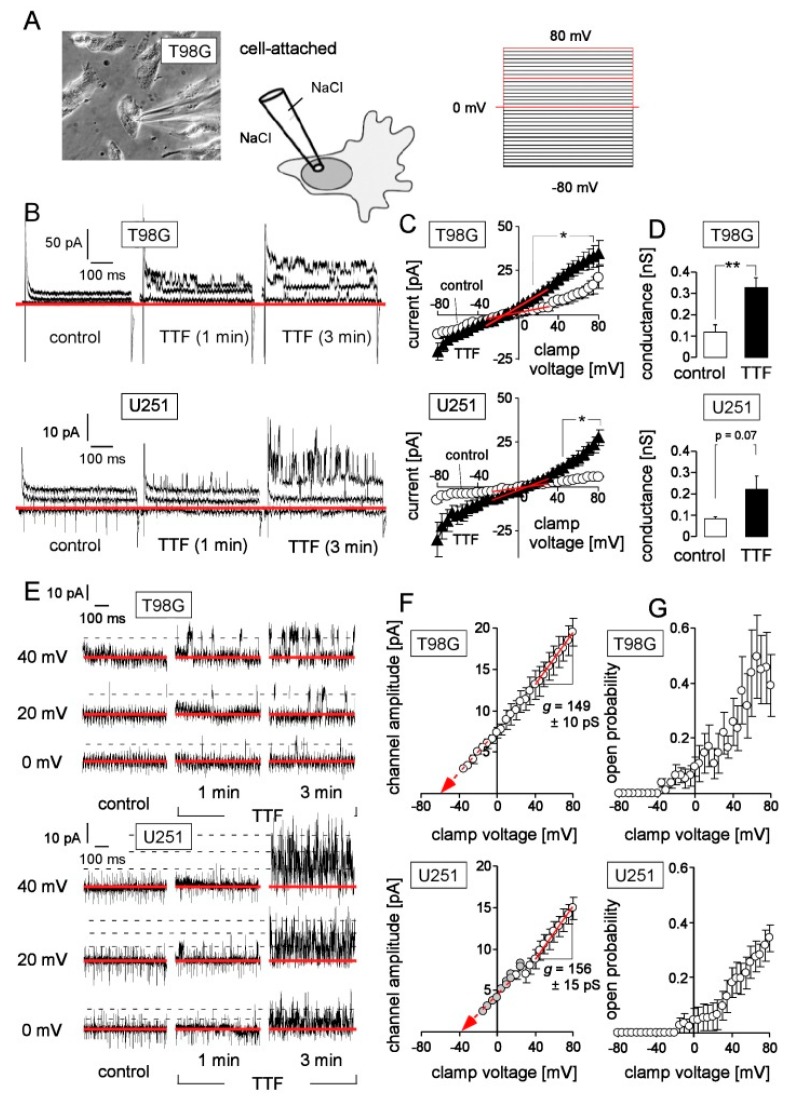
TTFields activate BK K^+^ channels. (**A**) Macroscopic cell-attached currents were recorded from T98G (micrograph on the left) and U251 cells with the patch-clamp technique using a physiological NaCl-rich extracellular solution in bath and pipette (middle). Currents were elicited by 33 square pulses (700 ms each) to voltages between −80 and +80 mV delivered from 0 mV holding potential in 5 mV increments as shown by the voltage-clamp pulse protocol on the right. (**B**) Cell-attached current tracings recorded as in (**A**) from a T98G (top) and a U251 cell (bottom) before (left), after 1 min (middle) and 3 min (right) TTFields (200 kHz, 2.5 V/cm) application. As illustrated by the red lines in the pulse protocol (**A**, right), only currents elicited by voltage steps to 0, +40 and +80 mV are shown (red lines in (**B**) indicate the zero current). (**C**) Relationship between the mean (±SE; *n* = 10–11) macroscopic cell-attached currents and the clamp voltage in control (0 V/cm, open circles) and TTFields (2.5 V/cm)-treated (closed triangles) T98G (*n* = 10, top) and U251 cells (*n* = 6, bottom). (**D**) Mean (±SE;) conductance of the clamped membrane area in T98G (top) and U251 cells (bottom) as calculated between −30 and +30 mV clamp voltage from the data in (**C**, red lines) by linear regression. * and ** in (**C**, and **F**, top) indicate *p* ≤ 0.05 and *p* ≤ 0.01, respectively, Welch-corrected *t*-test. The square brackets in (**C**) mark the voltage range, where control and TTFields-stimulated cell-attached currents differ significantly; the number in (**D**, bottom) indicates the *p* value. (**E**) Cell-attached current tracings recorded in a T98G (top) and a U251 cell (bottom) at 0, +20 and +40 mV clamp voltage before (control) and after 1 and 3 min of TTFields exposure disclosing TTFields-induced unitary outward current transitions (red lines and dashed lines indicate zero current and distinct current levels of up to three simultaneously open channels, respectively). (**F,G**) Relationship between channel amplitude (**F**) and estimated open probability (**G**) of the unitary current transitions in T98G (top) and U251 cells (bottom). Shown are mean (±SE, *n* = 3–5, open symbols) or individual data (grey symbols). The red lines and red arrows in (**F**) depict the clamp voltage range used to calculate the single channel conductance (*g*) by linear regression and extrapolate to the likely reversal potential, respectively.

To test for functional significance of this TTFields-induced rise in _free_[Ca^2+^]_i_, functionality of Ca^2+^-activated K^+^ channels in the plasma membrane was monitored shortly before and directly after TTFields application (2.5 V/cm for 1–3 min) by continuous cell-attached patch-clamp recording with physiological extracellular NaCl solution in bath and pipette (Figure 3A). 

Due to the experimental set-up with an electrical line from the function generator outside the Faraday cage to the TTFields applicator electrodes that were placed closely to the recorded cells inside the Petri dish, a high 50 Hz ripple superimposed the cell-attached currents. This rendered it impossible to resolve unitary current transitions generated by low or intermediate conductance ion channels. However, the “macroscopic” cell-attached currents as a measure of overall channel activity of the recorded membrane area could be analyzed. As a result, TTFields induced in both glioblastoma lines an instantaneous increase in macroscopic cell-attached outward currents at positive clamp voltages (Figure 3A–C). Moreover, in the range of the physiological membrane potential (i.e., at 0 mV clamp voltage, see below), TTFields increased (T98G cells) or showed a trend to increase (U251) the membrane conductance of the clamped membrane area (Figure 3D) suggesting that TTFields may interfere with the physiological electrosignaling of glioblastoma cells.

Remarkably, the macroscopic currents in Figure 3B (middle, right) disclose large unitary current transitions that could be characterized in more detail (Figure 3E–G). Figure 3E shows in higher power cell-attached current tracings at three clamp voltages (0, +20 and +40 mV) before (left) and after 1 min (middle) or 3 min (right) of TTFields application in T98G (top) and U251 cells (bottom). In both glioblastoma lines, TTFields stimulated the activity of a channel with a conductance in the range of 150 pS as deduced from the channel amplitude/voltage (I/V) relationships in Figure 3F. In addition, the I/V curves in this figure extrapolate (red arrow) to a reversal potential (i.e., where the curve crosses the *x*-axis) of around −60 mV in T98G cells (Figure 3F, top) and around −40 mV clamp voltage in U251 cells (Figure 3F, bottom). In cell attached mode when recorded with a physiological (i.e., 5 mM K^+^-containing) NaCl pipette and bath solution, the (negative) physiological membrane potential remains unaltered and applies on top of the clamp voltage across the recorded membrane. Thus, the actual reversal potentials of the I/V curves in Figure 3F are more negative than those deduced from the clamp voltage. This is important since it suggests that the actual reversal potential of the TTFields-activated channel is close to the highly negative K^+^ equilibrium potential indicating its K^+^ selectivity.

An estimation of the open probability (P_o_) of this high conductance K^+^ channel (Figure 3G) further suggests for both glioblastoma lines a depolarization-induced activation of the channel and an increase in its P_o_ with increasing positive voltage. Notably, the P_o_/voltage relationship (Figure 3G, bottom) as well as the I/V curve (Figure 3F, bottom) of U251 cells were shifted by some +20 mV when compared to the situation in T98G cells (Figure 3F,G, top). This can be explained with a higher negative physiological membrane potential in U251 as compared to T98G cells since the higher the physiological membrane potential the more positive clamp voltage is required to reach the activation voltage of the channel. Most importantly, K^+^ selectivity, high unitary conductance, and voltage dependence of the TTFields-induced channel were identical to the characteristics of paxilline-sensitive BK K^+^ channels reported earlier to be highly expressed in T98G and U251 cells [45].

The I/V curves in Figure 3C especially in U251 cells also show a trend to TTFields-stimulated inward currents which, however, did not reach statistical significance. The underlying single channel activity could not be resolved because of the above mentioned limitations of our experimental set up. Along those lines, inward current transitions generated by Ca^2+^ channels such as low conductance voltage-gated L-type channels (e.g., upon depolarization from 0 mV clamp voltage, i.e., from the physiological membrane potential, to +40 mV clamp voltage) could also not be resolved. The same holds true for IK_Ca_ (KCa3.1, SK4)-mediated outward current transitions. Nevertheless, our observations suggest that the observed TTFields-induced increase in _free_[Ca^2+^]_i_ is functionally relevant and sufficient to activate BK K^+^ channels.

**Figure 4:**
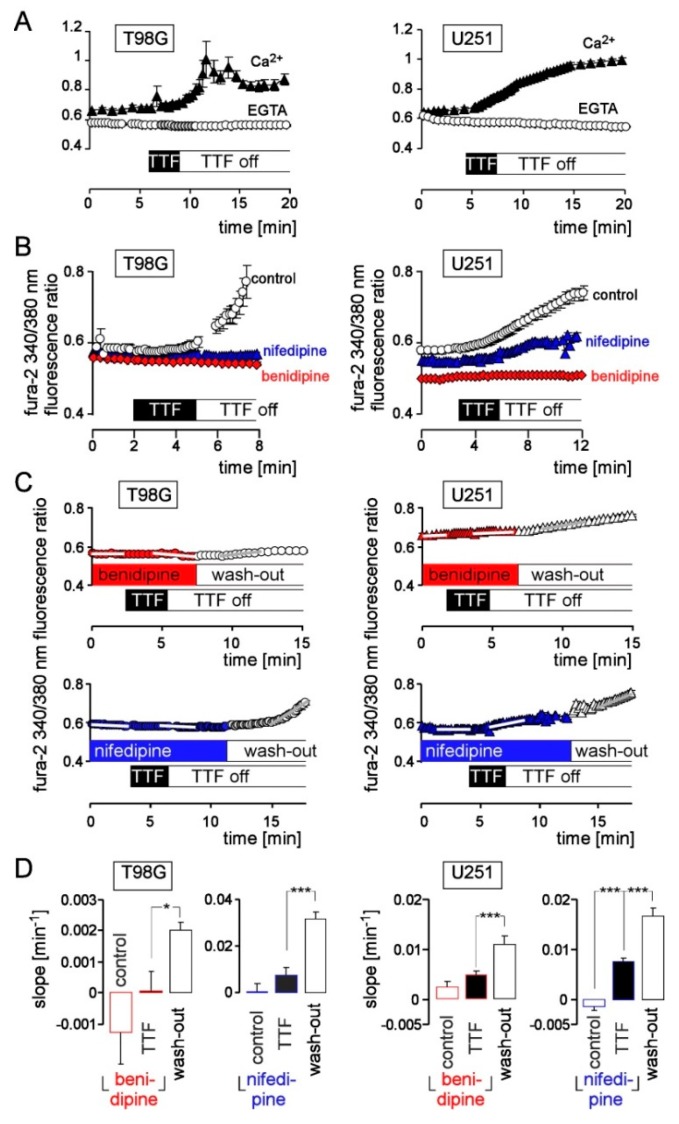
TTFields induce a dihydropyridine-sensitive Ca^2+^-entry in human glioblastoma cells. (**A**) Time course of TTFields (200 kHz, 2.5 V/cm, 3 min)-induced changes of mean (±SE; *n* = 8–20) fura-2 340/380 nm fluorescence ratio as recorded in T98G (left) and U251 (right) cells with Ca^2+^ (1 mM)-containing NaCl-solution (closed triangles) and EGTA (0.6 mM)-buffered Ca^2+^-free NaCl-solution (open circles). (**B**) Mean (±SE; *n* = 13–20) fura-2 340/380 nm fluorescence ratio recorded in T98G (left) and U251 cells (right) before, during, and after application of TTFields (200 kHz, 2.5 V/cm, 3 min) during continuous superfusion with Ca^2+^-containing NaCl-solution (open circles), Ca^2+^-containing NaCl solution further containing 1 µM benidipine (red triangles) or 1 µM nifedipine (blue diamonds). (**C**) Mean (±SE; *n* = 13–39) fura-2 340/380 nm fluorescence ratio recorded in T98G (left) and U251 cells (right) before, during, and after application of TTFields (200 kHz, 2.5 V/cm, 3 min) during continuous superfusion with benidipine (1 µM, top, red symbols) or nifedipine (1 µM, bottom, blue symbols)-containing NaCl-solution and after wash-out of the inhibitors (open symbols). (**D**) Mean (±SE; *n* = 34–63) slope (as indicated by white lines in (**C**)) of the fura-2 340/380 nm fluorescence ratio changes before (control), at the end and shortly after TTF-application (middle) both in the presence of benidipine (red) or nifedipine (blue) as well as after wash-out of the inhibitors. * and *** in (**D**) indicate 3*p* ≤ 0.05 and 3*p* ≤ 0.01, respectively, (Welch)-corrected *t*-test and Bonferroni correction for 3 pairwise comparisons.

Next, we characterized the TTFields-induced _free_[Ca^2+^]_i_ rise in further fura-2 experiments by analyzing its dependence on extracellular Ca^2+^ and its sensitivity to the Ca^2+^ channel inhibitors benidipine and nifedipine (1 µM, both). In both glioblastoma lines, decrease of extracellular Ca^2+^ by superfusion with Ca^2+^-free, EGTA-chelated NaCl solution (Figure 4A) or application of the L-, N-, T-type Ca_v_ channel blocker benidipine in Ca^2+^-containing superfusate (Figure 4B,C,E)) abolished the TTFields-induced rise in _free_[Ca^2+^]_i_. In contrast to benidipine, the L-type Ca_v_ inhibitor nifedipine abolished only in T98G cells the TTFields-induced _free_[Ca^2+^]_i_ rise completely while conferring only a partial blockage in U251 cells (Figure 4B,D,E). Notably, _free_[Ca^2+^]_i_ started to increase for several minutes upon wash-out of benidipine and nifedipine 2 min and 5 min, respectively, after switching off the TTFields stimulation (Figure 4C). Together, those experiments suggest a TTFields-induced Ca^2+^ entry via the activation of a nifedipine-sensitive L-type Ca_v_ channels in T98G and U251 cells and via an additional benidipine-sensitive and nifedipine-insensitive pathway in U251 cells. They further suggest that the TTFields effect on _free_[Ca^2+^]_i_ is not simply due to electroporation of the plasma membrane. Moreover, the inhibitor wash-out experiments demonstrate that the increase in _free_[Ca^2+^]_i_ that sustains for several minutes after end of the trigger is due to a sustained Ca^2+^ entry.

**Figure 5:**
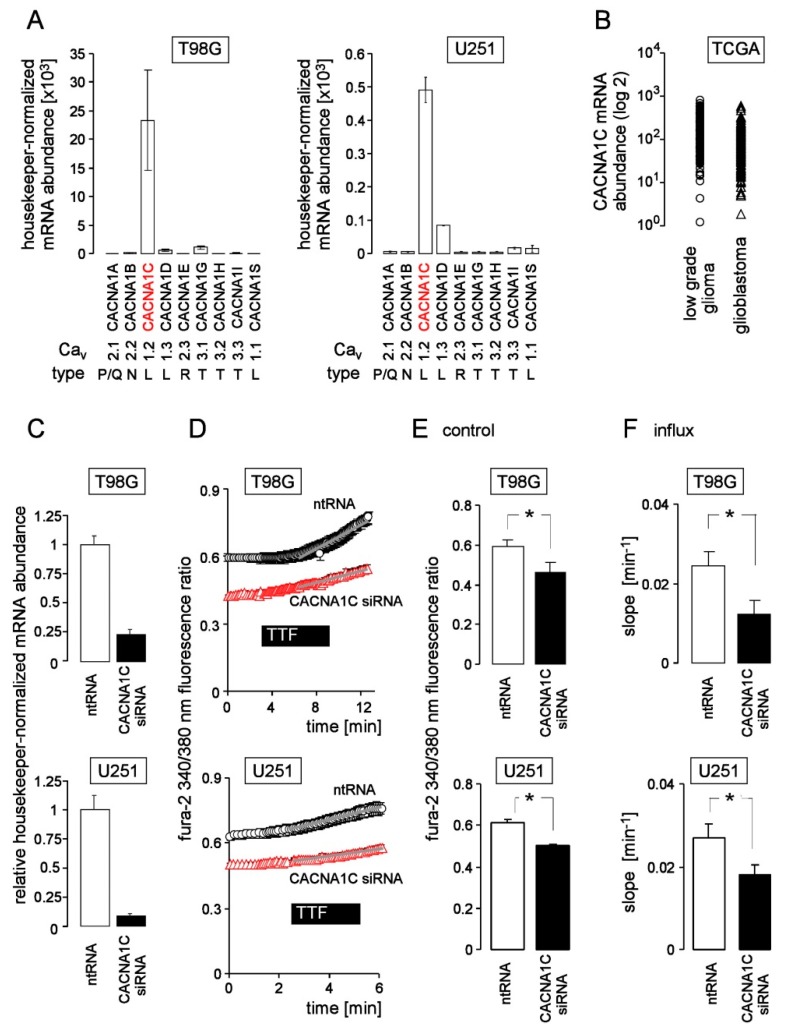
The voltage-gated L-type Ca^2+^ channel CACNA1C (Ca_v_1.2) contributes to the TTFields-induced Ca^2+^ entry. (**A**) Mean (±SE; *n* = 3) housekeeper-normalized mRNA abundances [×10^3^] of P/Q-, N-, L-, R- and T-type Ca_v_ channels CACNA1A, -1B, -1C, -1D, -1E, -1G, -1H, -1I, and -1S (as indicated) in T98G (left) and U251 (right) cells as determined by real-time RT-PCR indicating expression of CACNA1C in both human glioblastoma lines. (**B**) CACNA1C mRNA abundances (log2 RNA seq data) of human low grade glioma (left) and glioblastoma (right) specimens vary considerably between individual tumors. The provisional Glioblastoma-Multiforme and Lower-Grade-Glioma TCGA databases (http://cancergenome.nih.gov/) were queried for CACNA1C mRNA abundance in the tumor specimens via the cBIOportal Web resource [46,47]. (**C**) Mean (±SE; *n* = 3) relative housekeeper-normalized abundance of CACNA1C mRNA in T98G (top) and U251 (bottom ) cells 48 h after reverse transfection with CACNA1C-siRNA (closed bars) or non-targeting RNA (ntRNA, open bars). (**D**) Mean (±SE; *n* = 6–19) fura-2 340/380 nm fluorescence ratio of CACNA1C siRNA (red triangles)- or ntRNA-transfected (open circles) T98G (top) and U251 cells (bottom), before, during and after application of TTFields (200 kHz, 2.5 V/cm, 3–5 min). (**E,F**) Mean (±SE; *n* = 6–19) control ratio (**E**) and TTFields-induced increase in (**F**) fluorescence ratio of CACNA1C siRNA (red bars)- or ntRNA-transfected (open bars) T98G (top) and U251 cells (bottom). * indicates *p* ≤ 0.05, Welch-corrected *t*-test).

Further on, we analyzed the expression of potential benidipine targets in glioblastoma. To this end, we assessed the abundance of mRNA encoding Ca^2+^ channels in our two human glioblastoma cell lines and in glioma resection specimens by RT-PCR and by querying the TCGA low grade glioma and provisional glioblastoma data bases, respectively. Among all tested mRNAs, the mRNA of the L-type channel CACNA1C (Ca_v_1.2) was most abundant in T98G and U251 cells (Figure 5A). In T98G, housekeeper-normalized CACNA1C mRNA abundance exceeded that of U251 cells by almost factor of 50. Similarly, CACNA1C mRNA abundance varied considerably in resection specimens of low grade and high grade glioma (Figure 5B). Together, these data suggest CACNA1C is expressed in glioblastoma albeit at variable extent. To test for functional significance of this channel, the effect of CACNA1C knockdown (Figure 5C–F) on control and TTFields-modulated _free_[Ca^2+^]_i_ in T98G and U251 cells was determined by fura-2 Ca^2+^ imaging. As shown in Figure 5D,E, transfection with CACNA1C siRNA decreased resting _free_[Ca^2+^]_i_ suggesting constitutive activity of Ca_v_1.2 in both cell lines. Moreover, CACNA1C siRNA lowered the slope of the TTFields-induced _free_[Ca^2+^]_i_ rise in T98G and U251 cells (Figure 5D,F) pointing to an involvement of Ca_v_1.2 in here.

**Figure 6:**
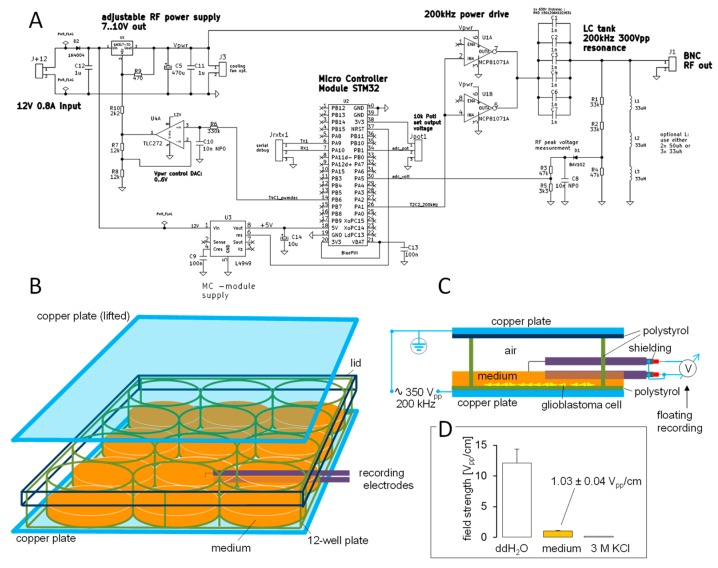
Cell culture TTFields applicator. (**A**) Circuitry of applicator. Frequency and amplitude of the 200 kHz, 300 Vpp TTFields signal is controlled by a “STM32 blue pill” micro controller module (MCM). Software was developed with the free ARDUINO programming environment. A 200 kHz digital clock is generated by the MCM internal programmable timers. This clock is amplified by a power drive stage that delivers sufficient current and voltage to drive a 200 kHz series LC resonance tank. LC tank resonance transforms the digital 7 to 10 V peak to peak drive signal into a clean sine wave of 200 kHz and up to 350 V peak to peak at the RF output. Amplitude can be set by a potentiometer which controls the supply voltage of the LC-tank power drive. (**B**–**D**) Calibration of the applicator. TTFields (maximal output) are applied capacitively via two electrically isolated copper foils mounted below and above a 12-well cell culture plate. The electric field strength applying to the cells was determined under the chosen experimental conditions by the use of two shielded electrodes that were inserted laterally in a central well of a 12-well plate measuring chamber filled with 1 ml culture medium in every well (**B**,**C**). For quality control of this measurement, the culture medium of the recorded well was replaced by media with lower (_dd_H_2_O) and higher (3 M KCl) dielectric constant. As a result, electric field strength (maximal output) in cell culture medium was the range of 1 V/cm. Moreover, decreasing (_dd_H_2_O) or increasing the permittivity (3 M KCl) increased and decreased, respectively, the electric field strength accordingly (**D**, data are means ±SE, *n* = 3–4). In particular, electric field strength in _dd_H_2_O was about 12-times that of plasma-isotone cell culture medium (**D**) which suggests a relative dielectric constant of around 1000 for the cell culture medium similar to the value reported for human plasma in this wavelength range [48]. The TTFields application (5–7 days) was performed in a normal humidified 37° C cell culture incubator in 5% CO_2_ atmosphere. Continuous TTFields application did not increase the temperature of the cultured cells. Temperature recording during up to 24 h of TTFields (maximal output) with a thermo-resistor directly placed in the fluid column of the cell-culture well did not disclose any TTFields-associated temperature increase (36.4 ± 0.17 °C, *n* = 5) as compared to control recording in the same set-up with switched-off TTFields (36.5 ± 0.07 °C, *n* = 3).

**Figure 7:**
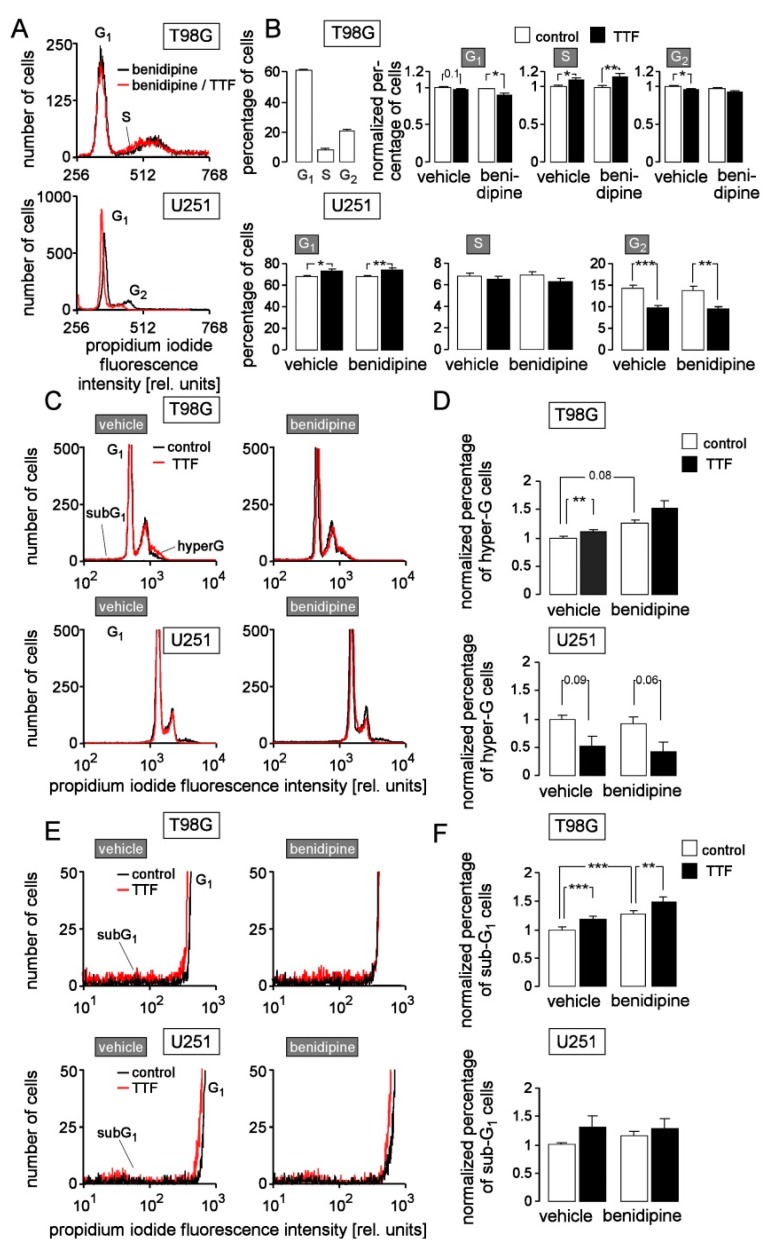
TTFields stimulate a G_1_ or S cell cycle arrest in T98G and U251 cells and an increase in hyper-G and sub-G_1_ populations in T98G cells. Benidipine (1 µM) tends to augment or significantly augments these latter effects. (**A**,**C**,**E**) Histograms recorded by flow cytometry showing the DNA content (Nicoletti staining with propidium iodide) of T98G (top) and U251 cells (bottom) treated for 7 days with TTFields of 0 (black) or 1 V/cm (red) field strength and benidipine (0 or 1 µM). (**B**,**D**,**F**) Mean (±SE; *n* = 12–22) percentage or normalized percentage of cells residing in G_2_, S or G_1_ phase of cell cycle (**B**, upper line, 2nd to 4th bar diagram and lower line; the bar diagram in the upper left gives the absolute values for the control situation in T98G), cells with elevated DNA content (hyper-G population, **D**; absolute values in the control situation were 7.0 ± 0.6% for T98G and 3.9 ± 0.5% for U251), and dead cells (sub-G_1_ population, **F**; absolute values in the control situation were 2.8 ± 0.3% for T98G and 6.3 ± 0.4% for U251 ). *, ** and *** indicate 3*p* ≤ 0.05, 3*p* ≤ 0.01, and 3*p* ≤ 0.001, respectively, Welch-corrected *t*-test and Bonferroni correction for three pairwise comparisons (vehicle/control vs. benidipine/control, vehicle/control vs. vehicle/TTFields, and benidipine/control vs. benidipine/TTFields). Numbers in (**B**, upper line, 2nd diagram) and (**D**) indicate *p* values.

To study the interaction of TTFields with cell biology, we developed a cell culture TTFields applicator that applied 200 kHz sine wave electrical fields to a multi-well plate (Figure 6A). The field (1 V/cm peak-to-peak amplitude) was injected perpendicular to the cell layer in a capacitive manner via electrically isolated copper foils mounted below the bottom and above the lid of the multi-well plate (Figure 6B–D). To identify effects of TTFields on cell cycle and the role of Ca_v_1.2 herein, we co-treated T98G and U251 cells for 5–7 days with TTFields (0 or 1 V/cm) and benidipine (0 or 1 µM) and determined the cellular DNA content by propidium iodide staining in flow cytometry thereafter. In T98G, TTFields showed a tendency to decrease G_1_ population and significantly increased S and decreased G_2_ populations (Figure 7A top and Figure 7B, upper row) suggestive of a TTFields-induced S phase arrest. Benidipine alone did not affect cell cycle distribution in T98G cells but augmented the TTFields effect on G_1_ and S populations (Figure 7A top and Figure 7B, upper row). In contrast, TTFields increased G_1_ and decreased G_2_ population of U251 cells (Figure 7A bottom and Figure 7B, lower row) indicative of a TTFields induced G_1_ arrest. Unlike T98G, benidipine had no effect on cell cycle distribution of U251 cells (Figure 7B, lower row).

To further identify TTFields-caused asymmetric cell division, hyper-G population (i.e., cells with DNA content larger than normal diploid cells) was quantified as a measure of chromosome aneuploidy. In T98G, benidipine tended to elevate and TTFields increased hyper-G population, and both effects seemed to be additive (Figure 7C,D, top each). In U251, by contrast, TTFields tended to decrease hyper-G population while benidipine had no effect (Figure 7C,D, bottom each). Finally, sub-G_1_ population was analyzed as a measure of dying or dead cells with degraded DNA. In T98G, TTFields and benidipine additively induced cell death (Figure 7E,F, top each). In U251, in contrast, neither TTFields nor benidipine evoked cell death (Figure 7E,F, bottom each).

**Figure 8:**
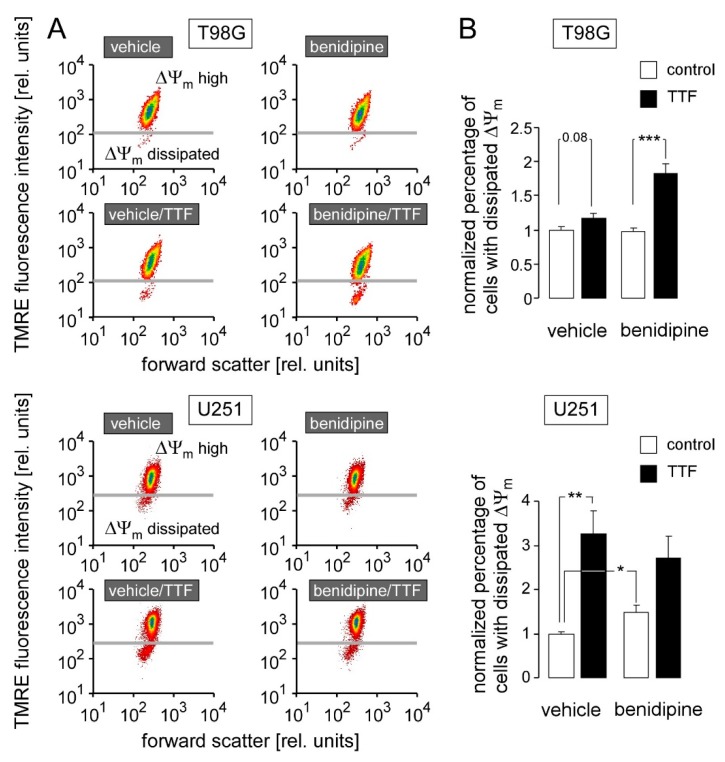
Benidipine augments TTFields-induced breakdown of the inner mitochondrial membrane potential (ΔΨ_m_) in T98G cells. (**A**) Dot plots showing forward scatter and TMRE fluorescence intensity of T98G (top) and U251 cells (bottom) treated for 7 day with 0 (control) or 2.5 V/cm TTFields (200 kHz) in the absence (vehicle) or presence of benidipine (1 µM). (**B**) Mean (±SE; *n* = 12–22) normalized percentage of T98G (top) and U251 (bottom) cells with dissipated ΔΨ_m_ after 5–7 days of treatment with 0 (control) or 1 V/cm TTFields (TTF, 200 kHz) and 0 or 1 µM benidipine (absolute values of the control situation were 4.5 ± 1.0% for T98G and 4.3 ± 1.3% for U251). *, ** and *** indicate 3*p* ≤ 0.05 and 3*p* ≤ 0.01, respectively, Welch-corrected *t*-test and Bonferroni correction for three pairwise comparisons (vehicle/control vs. benidipine/control, vehicle/control vs. vehicle/TTFields, and benidipine/control vs. benidipine/ TTFields). Number in (**B**, top) indicates *p* value.

To characterize potential death pathways, we determined the inner mitochondrial membrane potential (∆Ψ_m_) by TMRE staining in flow cytometry in T98G and U251 cells treated with TTFields (0 or 1 V/cm) and benidipine (0 or 1 µM) using time schedules identical to those applied for cell cycle analysis. In T98G, TTFields showed a trend to induce ∆Ψ_m_ dissipation as indicated by low TMRE staining while benidipine alone had no effect. In combination, however, TTFields and benidipine evoked a doubling of cell population with dissipated ∆Ψ_m_ suggesting their synergistic action (Figure 8A,B top each). In U251, in contrast, TTFields and benidipine in each case alone stimulated ∆Ψ_m_ dissipation (3-fold and 1.5-fold, respectively), effects that were not additive in combined treatment (Figure 8A,B bottom each). Together, these data suggest that TTFields alone (U251) or in combination with benidipine (T98G) may trigger intrinsic apoptosis.

**Figure 9:**
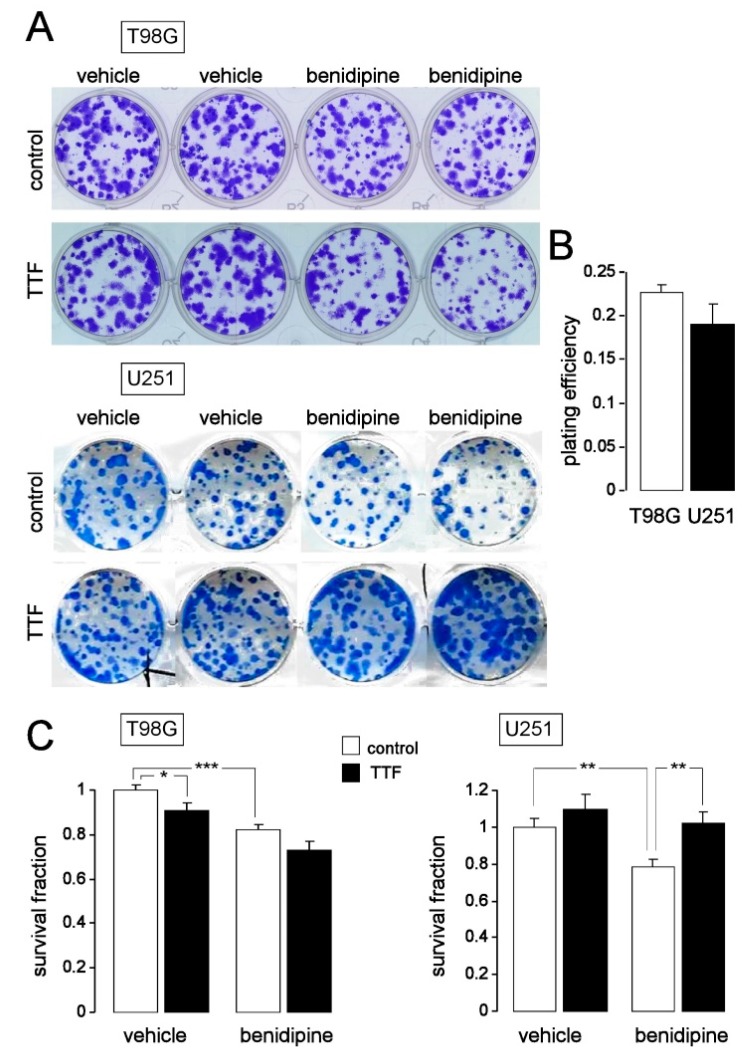
TTFields reduce clonogenic survival of T98G but not of U251 cells. (**A**) Pre-plating colony formation of T98G (top) and U251 (bottom) cells after treatment with 0 V/m (control) or 1 V/m TTFields field strength and co-incubation with vehicle alone or benidipine (1 µM) for 7 days. Shown are cut-outs of 6-well plates with Coomassie-stained T98G (top) and U251 (bottom) colonies. (**B**) Mean (±SE, *n* = 16–22) plating efficiency of T98G and U251 cells. (**C**) Mean (±SE, *n* = 16–24) survival fractions of T98G (left) and U251 cells (right) after treatment with 0 V/m (control) or 1 V/m TTFields strength and co-incubation with vehicle alone or benidipine (3 µM) for 7 days. Data were obtained by pre-plating colony formation assay as shown in (**A**). *, ** and *** indicate 3*p* ≤ 0.05, 3*p* ≤ 0.01, and 3*p* ≤ 0.001, respectively, Welch-corrected *t*-test and Bonferroni correction for three pairwise comparisons (vehicle/control vs. benidipine/control, vehicle/control vs. vehicle/TTFields, and benidipine/control vs. benidipine/TTFields).

Finally, we defined whether or not TTFields-induced cell death may lower clonogenic survival which is the most relevant endpoint in oncology with regard to tumor relapse after therapy. Pre-plating colony formation assay (5–7 days of TTFields with 0 or 1 V/cm and co-incubation with 0 or 3 µM benidipine followed by 10–14 days of post-incubation in the continuous presence of benidipine until formation of colonies, Figure 9A,B) suggest that both, TTFields- and benidipine monotherapies, attenuated clonogenic survival of T98G cells. In combination, the effects of both therapies tended to be additive (Figure 9A, top and Figure 9C, left). In U251, in sharp contrast, TTFields rather increased clonogenic survival and abolished the inhibitory effect of benidipine (Figure 9A, bottom and Figure 9C, right). In summary, our experiments suggest that TTFields modulate Ca^2+^ signaling in two human glioblastoma cell lines which involves long-lasting activation of L-type Ca_v_1.2 (CACNA1C) and most probably further Ca_v_ channels and which was completely suppressed by Ca^2+^ channel inhibitor benidipine. Cell line-dependently, TTFields induce S (T98G) or G_1_ cell cycle arrest (U251), aneuploidy, and DNA degradation (T98G), triggered intrinsic apoptosis (U251), and decreased clonogenic survival (T98G). Importantly, in T98G cells which exhibited about 50 times higher CACNA1C mRNA abundance than U251, but not in U251 cells, benidipine aggravated TTFields-triggered cell cycle arrest, intrinsic apoptosis and DNA degradation and showed a tendency to act additively to TTFields on induction of aneuploidy and attenuation of clonogenic survival. In U251, benidipine alone triggered intrinsic apoptosis and decreased clonogenic survival. The latter was reversed by TTFields.

## 4. Discussion

In our study, TTFields impaired clonogenicity of T98G but not of U251 human glioblastoma cells indicating that individual glioblastoma may respond differentially to this electric field therapy. The inhibition of clonogenic survival of T98G cells by 5–7 days of TTFields was below 10%. At a first glance, this effect seems to be too low to become tumor biologically relevant. However, one has to take into account that field direction (perpendicular and not parallel to the cell layer and field strength (1 V/cm instead of 3 V/cm) did not match the reported [4] optimal settings due to technical limitations of our set-up. This might hint to an underestimation of the TTFields effect by our experiments. In addition, overall treatment time of several months might lead to significantly larger effects on glioblastoma cell death and clonogenic regrowth compared to the one week application of TTFields for our in vitro experiments.

The 6-years follow up of the multicentric randomized prospective clinical trial analyzing temozolomide vs. temozolomide plus TTFields maintenance therapy suggests that in particular glioblastoma patients with methylated MGMT (O-6-methylguanine-DNA methyltransferase) promotor in the tumor benefit from TTFields therapy [2]. This might suggest that efficacy of TTFields increases with decrease of MGMT activity. In our experiments, however, the TTFields more responding line T98G has been reported to express higher MGMT activity [49] than the low TTFields responder U251 [50]. Alternatively, one might speculate that TTFields which is applied concurrently to temozolomide maintenance therapy becomes especially effective in MGMT promotor-methylated, temozolomide-sensitive cells pre-damaged by the chemotherapy. As a matter of fact, temozolomide and TTFields have been reported to synergistically accelerate cell death of irradiated glioblastoma cells in vitro, however, independently of MGMT status [51]. Additive anti-proliferative effects of temozolomide and TTFields have also been reported for temozolomide-sensitive and resistant glioblastoma stem cell-enriched primary cultures [52]. Along those lines, TTFields have been shown in vitro to improve the efficacy of radiotherapy in glioblastoma [23] and non small cell lung cancer cells [14] possibly by impairing repair of DNA double strand breaks [14]. Combined, this might suggest that TTFields are especially efficient in combination with other cytotoxic anti-cancer therapies such as radiotherapy or chemotherapy and enhance their effect as has been described for moderate locoregional hyperthermia [53]. As a matter of fact, combining mitotic checkpoint inhibition with TTFields in vitro synergistically enhance apoptotic cell death in glioblastoma cells [54].

The reported interference with DNA repair by TTFields [14] also suggests that TTFields exert additional cellular effects beyond impairment of mitotic spindle formation and cytokinesis as proposed cellular mechanisms of anti-neoplastic TTFields action (see Section 1). Evidence for the latter two came in our study from the TTFields-induced increase in T98G cell population with hyper-G DNA content as a measure of aneuploidy. Beyond that, our study identified benidipine-sensitive Ca^2+^ channels that comprise L-type CACNA1C (Ca_v_1.2) channels as further TTFields targets. The dihydropyridine calcium channel blocker benidipine acts on L-, T-, and N-type channels. Like in the present study, alternating current electromagnetic fields (ac-EMF) such as microwave or Wi-Fi have been demonstrated in several independent studies to activate Ca^2+^ entry pathway in various cell types. Notably, these pathways were in part sensitive to inhibitors of voltage-gated Ca^2+^ channels suggesting that the ac-EMF sensitivity of voltage-gated Ca^2+^ channels is a general phenomenon (for review see [33]). Particularly striking was the fact that in our experiments Ca_v_-mediated Ca^2+^ influx outlasted TTFields stimulation by far suggesting a constitutive activity of Ca_v_ channels. As a matter of fact, such constitutive activity has been reported in smooth muscle cells for L-type Ca^2+^-channels. This constitutive activity and functional clustering of channels reportedly are mediated by protein kinase C (PKC) and determine the resting _free_[Ca^2+^]_i_ in these cells [55]. Along those lines, pulsed electrical field induced contraction of feline esophageal smooth muscle cells reportedly requires both, extracellular Ca^2+^ and PKC activity [56]. This might suggest that similar processes contribute to the long-lasting Ca^2+^ entry in TTFields-treated glioblastoma cells.

Taking into account that cell cycle [57], cell migration [38,45,57], brain infiltration [40], cell death programming [57], DNA repair and radioresistance [42,57] of glioblastoma cells have been demonstrated to be regulated by Ca^2+^-dependent signaling pathways [58] an interference of TTFields with glioblastoma biology can be expected. In line with this assumption, the present study demonstrated that TTFields indeed interfered with all cell biological parameters tested.

Of note, the voltage-gated Ca^2+^ channel blocker benidipine did not revert the observed cellular TTFields effects indicating that these effects most probably were not induced by TTFields-triggered benidipine-sensitive Ca^2+^ influx. Rather, benidipine aggravated (or showed the tendency to increase) the TTFields effects in T98G cells. Unexpectedly, such a concerted action of benidipine and TTFields was not observed in U251 cells although both glioblastoma lines exhibited comparable TTFields-induced Ca^2+^ entries that were both completely blocked by benidipine (see Figure 4). In contrast to T98G, TTFields decreased aneuploidy and rather increased than decreased clonogenic survival in U251 again illustrating that individual glioblastomas may respond differentially to TTFields.

Irrespective of its effect in combination with TTFields, benidipine alone induced in the present study in both glioblastoma lines either DNA degradation (T98G) or ∆Ψ_m_ dissipation (U251) and lowered clonogenic survival (both lines) indicating its anti-neoplastic action. Among the known benidipine targets, mRNA encoding the L-type Ca^2+^ channel CACNA1C (Ca_v_1.2) was most abundant in both glioblastoma lines strongly suggesting that the observed benidipine effects were mediated at least in part via inhibition of CACNA1C. The functional significance of CACNA1C for the Ca^2+^ signaling of both glioblastoma cell lines can be deduced from the decline in resting Ca^2+^ concentration (i.e., control _free_[Ca^2+^]_i_, see Figure 5E) upon knockdown of CACNA1C.

The notion of a presumable general anti-neoplastic function of benidipine in glioblastoma in concert with the observed TTFields stimulation of a benidipine-sensitive target that probably counteract the TTFields effects (at least in the TTFields more responding glioblastoma cell line T98G) provides a rationale to combine alternating electric field therapy with Ca^2+^ antagonists even in tumors with unknown TTFields responsiveness. Benidipine, which is in clinical use in Japan and India (Coniel^®^; Kyowa Hakko Kirin Co., Ltd., Tokyo, Japan [59]) as anti-hypertensive drug is well tolerated. Tissue distribution in rats of orally applied benidipine indicates drug accumulation predominantly in digestive organs, mesenteric lymph nodes, liver, pancreas, urinary bladder, fat tissue, kidney and spleen [60]. The usually high penetrability of the glioblastoma blood barrier is expected to facilitate delivery of benidipine specifically to the glioblastoma while the blood brain barrier can be assumed to hamper distribution of benidipine in healthy brain parenchyma. Combined, this suggests that targeting of glioblastoma Ca^2+^ channels by benidipine or other FDA-approved Ca^2+^-antagonists such as nifedipine [61] seems to be clinically feasible.

## 5. Conclusions

TTFields act on several molecular targets/pathways thereby influencing Ca^2+^- and electrosignaling, cell cycle progression, programmed cell death, and clonogenic survival of glioblastoma cells. Concerning the most relevant parameter in oncology that is the clonogenic survival, TTFields responsiveness differed markedly between the two human glioblastoma lines tested suggesting that also in the clinical situation the benefit of TTFields therapy may vary considerably between individual glioblastoma patients. Knowledge of the underlying molecular mechanisms might be used for therapy stratification in the future or for pharmacological intervention and improvement of the tumoricidal TTFields effects. One such potential approach evolves from the present study that disclosed that Ca_v_ channel activity contributes to cellular stress response to TTFields and Ca_v_ inhibition may augment the TTFields effects.
